# Hospital Disaster Preparedness Tools: a Systematic Review

**DOI:** 10.1371/currents.dis.7a1ab3c89e4b433292851e349533fd77

**Published:** 2015-09-14

**Authors:** Esmail Heidaranlu, Abbas Ebadi, Hamid Reza Khankeh, Ali Ardalan

**Affiliations:** Health Management Research Center, Baqiyatallah University of Medical Sciences, Tehran, Iran; Behavioral Sciences Research Center (BSRC), Nursing Faculty of Baqiyatallah University of Medical Sciences, Tehran, Iran; Department of Health in Emergency and Disaster, University of Social Welfare and Rehabilitation Sciences, Tehran, Iran; Department of Clinical Science and education, Karolinska Institute, Stockholm, Sweden; Disaster and Emergency Health Academy, National Institute of Health Research, School of Public Health, Tehran University of Medical Sciences, Tehran, Iran; Harvard Humanitarian Initiative, Harvard University, Cambridge, USA

**Keywords:** disaster, Hospital preparedness, Measurement tool, Systematic review

## Abstract

Aim: Evaluating hospital disaster preparedness is one the best ways for hospital accreditation. The aim of this study was to evaluate the quality of outcome measure that offer the level of measurement, reliability and validity that are known as the ‘ psychometric properties’ of the current hospital disaster preparedness tools.

Methods: In total, 140 studies were retrieved. Studies which had been published from 2000 to 2014 and had used hospital disaster preparedness tools were appraised by using the PRISMA guideline. The content quality and the quality of the psychometric properties of the retrieved tools were assessed by using the World Health Organization Criteria for Hospital Preparedness as well as the COSMIN criteria.

Findings: Only 33 studies met the inclusion criteria. In total, eleven hospital disaster preparedness tools had been used in these 33 studies. These tools mainly focused on evaluating structural and non-structural aspects of hospital preparedness and paid little attention, if any, to the key functional aspect.

Conclusion: Given the paramount importance of evaluating hospital disaster preparedness and the weaknesses of current preparedness evaluation tools, valid and reliable tools should be developed by using experts’ knowledge and experience through the processes of tool development and psychometric evaluation.

Keywords: Hospital preparedness, Measurement tool, Disaster, Systematic review

## Introduction

Natural disasters have the potential to kill thousands of people in minutes. Moreover, many more people are killed during the following weeks and years due to the consequences of disasters 1. For example, in the Bam earthquake, the city was destroyed completely, >43,000 people were killed, and 30,000 were injured 2. During the past twenty years, natural disasters have affected more than three million families (i.e. at least 800 million people) worldwide and have cost more than $500 billion 3. Disasters affect all economic, political, and cultural infrastructures of afflicted communities 4 and inundate healthcare systems with huge numbers of victims for prolonged periods of time 5. Statistics show that about 3.4 billion people live in natural disaster hot spots 1.

Hospitals are among the healthcare centers whose prompt and efficient services can play a significant role in decreasing disaster mortality rate 6. Accordingly, hospitals should be designed and built in such a way that they can effectively manage all kinds of high-pressure crisis situations 7 . Zaboli et al (2006) noted that disasters cause healthcare systems and settings, particularly hospitals, serious disruption to health care services 8.

Effective disaster management necessitates having adequate disaster preparedness hospital disaster preparedness (HDP) 9 which is one of the major concerns of the World Health Organization 10. Numerous studies and attempts have been made worldwide for evaluating and improving HDP 11. But, there is still no standard and valid tool for evaluating it 12. Therefore, evaluation of studies that assess hospital disaster preparedness tools can provide useful data for researchers and users in selection of suitable tool 13.This study was conducted to evaluate the quality of outcome measure that offer the level of measurement, reliability and validity that are known as the ‘ psychometric properties’ of the current HDP tools.

## Materials and Methods

**Database and search strategies**

This systematic review study was conducted by using the Preferred Reporting Item for Systematic Reviews and Meta-analyses (PRISMA) guideline 14. Primarily, a study protocol was developed which consisted of formulating the study question, defining the inclusion criteria, developing a database search strategy, retrieving the relevant studies, extracting the relevant data, apprising the retrieved studies, synthesizing the data, and reporting them. We searched databases such as Google scholar, MEDLINE, Web of science, ProQuest, Science Direct, Ovid, Scopus, Cochrane, and CINAHL and retrieved articles published from 2000 to 2014. The search terms—which had been identified by consulting disaster management experts—included hospital, functional preparedness, natural disaster, operational preparedness, readiness, instrument, questionnaire, test, assessment, measurement, tool, and inventory. After conducting comprehensive search for the relevant articles, we also searched the reference lists of the retrieved articles for pinpointing the relevant documents. Given the abundance of the retrieved articles, we limited our search to Medical Subject Headings (MeSH).

**Inclusion and exclusion criteria**

The inclusion criteria were being published in English, being conducted by using qualitative, quantitative, or mixed method designs, dealing with either empirical or theoretical aspects of HDP, being conducted on human subjects, and being published in the area of medicine. The articles which did not have abstract as well as the duplicated ones were excluded. The first and the second authors separately read and reviewed the full texts of the retrieved articles to identify the factors and criteria that had been used for evaluating HDP. These activities helped us retrieve the most relevant articles and maintain the rigor of the study. Any disagreement between the two authors was resolved by consulting the third author 15. They also were requested to consider any risk of bias.

**Quality assessment scale**

We employed the four-point Consensus-based Standards for the selection of health Measurement Instruments (COSMIN) to appraise the retrieved articles regarding the psychometric properties of their data collection tools. The main psychometric properties of a tool according to the COSMIN are content, criterion, and construct validity, stability, internal consistency, responsiveness, and interpretability 15. The COSMIN is used to answer the following questions,

1. Is this a general or a specific tool?
2. How has this tool been developed?
3. How is the scoring preformed?
4. Has the tool been developed through the process of psychometric evaluation?
5. Which construct is measured by the tool?
6. Has the tool been developed by using a theoretical framework?
7. How is it completed?
8. Is it completed easily and conveniently?
9. Are complex statistical analyses needed for calculating the score of the tool?
10. Is it a contest-bound tool or an international one?

The psychometric properties of outcome measures include such things as level of measurement, reliability and validity 16 ^,^ 17 .Also, the psychometric of tools have shown the amount of accuracy and precision for users. valid and reliable tools provide authentic data to researchers 18.

Accordingly, we developed a checklist based on the COSMIN criteria for evaluating the quality of the retrieved tools. After data entry of three studies, we revised our checklist and used the revised one for evaluating the tools of other studies. The content quality of the tools was assessed in three structural, non-structural, and functional domains proposed by the Preparedness indexes of World Health Organization (Table 1).

**Figure:**
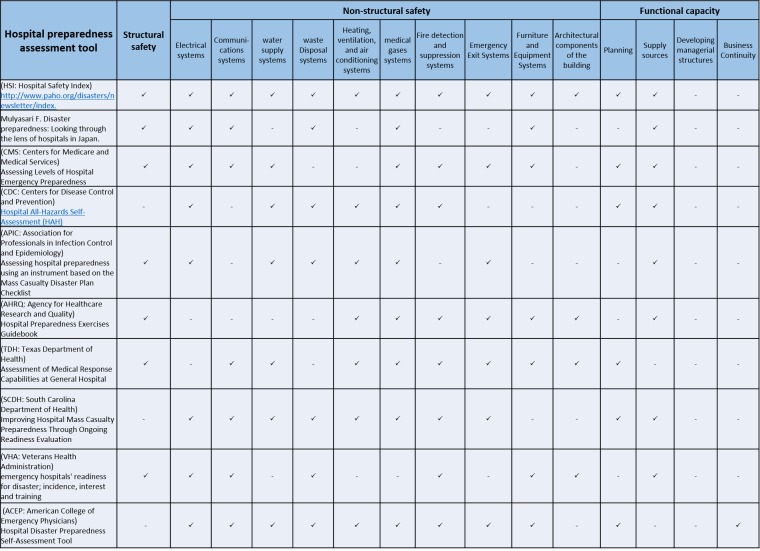
**Table 1: The content quality of the tools was assessed in three structural, non-structural, and functional domains**

## Findings

In total, 525 articles were retrieved (Figure 1) among which, 33 had reported using HDP tools. We carefully reviewed these 33 articles and found that only ten articles had referred to the psychometric properties of their tools as well as the structural, non-structural, and functional indicators of HDP. These ten articles were included in the final analysis (Table 1). Respectively, two studies had provided information concerning criterion and nine studies had provided information about construct validity, while all these ten studies had reported findings about the reliability of their tools. However, despite its importance, only three studies had evaluated and reported responsiveness (Table 2). This study carried low risk of bias because of its design and subject matter.

**The retrieval and selection process of articles in the literature review process:**
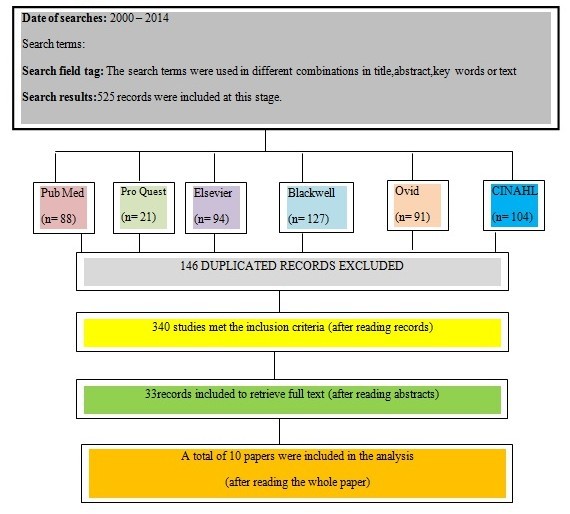

**Figure:**
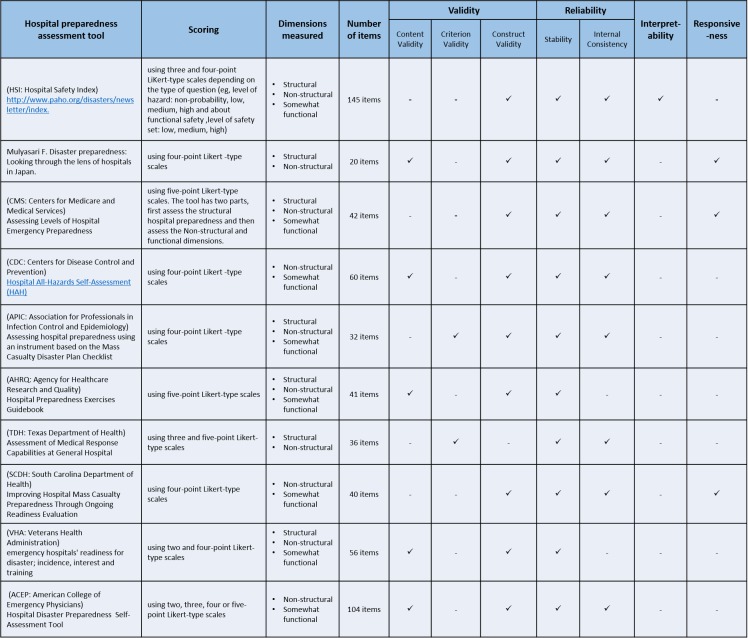
**Psychometric Evaluation of Hospital preparedness tools.**

## Discussion

The aim of systematic reviews on methodological researches is to identify their strengths and weaknesses and to highlight the necessity for conducting further studies for developing standardized instruments 13. Certainly, systematic reviews cannot provide absolutely precise and unbiased information about the accuracy and the precision of an instrument. However, these studies provide authorities with clear evidence for making wise decisions and form sound basis for further studies 19 ^,^ 20.

Nowadays, in the opinion of experts, Hospital Disaster Preparedness (HDP) is far beyond having a strong structures or modern high-tech equipment and includes suitable function in response during disasters 21. Disaster managers need valid and effective instruments for evaluating disaster preparedness of hospitals. However, there is no standardized, comprehensive instrument for this purpose and hence, various instruments have been developed and are used currently worldwide. Moreover, most of the current instruments are situation-specific or one-dimensional 3 ^,^ 20 ^,^ 22. The commonest tool for evaluating HDP is the World Health Organization Hospital Safety Index (HIS). This tool was developed by the Pan American Health Organization (PAHO) and the Disaster Mitigation Advisory Group (DIMAG) for evaluating hospital safety and preventing disaster-induced damages to healthcare centers and settings 11 ^,^ 23.However, the HIS does not provide objective measures for evaluating the functional domain and hospital preparedness 24.

In this systematic review study, in accordance with the guidelines of tool development, we used ten criteria for evaluating the psychometric properties of the current HDP tools. These ten criteria and the discussion of our evaluations are presented below.

1. Is this a general or a specific tool?

According to the standard criteria, appropriate tool(s) should be used for evaluating the preparedness of each hospital based on its main mission(s) 25.Some studies had provided data on the validity and the reliability of general disaster preparedness tools such as HIS 10.However, more specific and comprehensive tools are needed for properly evaluating the functional disaster preparedness of hospitals 26. Our findings revealed that none of the reviewed tools specifically evaluate different aspects of disaster preparedness. Instead, most of the tools generally evaluate the vulnerability of hospitals.

2. How has the tool been developed?

The best tools are the ones that have been developed through strictly adhering to the instrument development guidelines 27 ^,^ 28. Our findings revealed that various strategies had been used for developing HDP tools. Moreover, as none of the reviewed tools had been standardized, we could not compare them with each other. Most of the reviewed studies (CMS, APIC, and SCDH) had not provided precise information on the aim(s) of developing their tools, the target population, and the process of item generation. Moreover, most of them had not used disaster experts’ knowledge and experience for developing their tools.

3. How are the items responded to?

The process of responding to the items of a tool significantly contributes to the depth of the data that is obtained from it 29. Most of the reviewed tools used a four-point Likert-type scoring system. Other tools were scored dichotomously or by using two, three, or five-point Likert-type scales.

4. Has the tool been developed through the process of psychometric evaluation?

HDP tools should have acceptable psychometric properties, particularly content validity. The items of such tools need to be developed by considering functional disaster preparedness of hospitals 30. Content validity shows the extent to which the tool is capable of exactly measuring the intended construct. Generating the items of a tool by using the results of qualitative studies on experts and key informants can help ensure a great content validity 31 ^,^ 32. Beside content validity, HDP tools should also have an acceptable construct validity and reliability 33. Such tools need to be capable of identifying small changes and variations in the construct of interest. Accordingly, they should be developed by using strict psychometric evaluation guidelines. Our findings revealed that the most important weakness of the reviewed tools was limited data on their psychometric properties. Psychometric properties of the reviewed tools had not either been evaluated or reported. For instance, only few studies (HSI, CMS, SCDH) had provided data on the two important psychometric properties of responsiveness and interpretability. In addition, only half of the reviewed studies had reported content validity assessment. These studies had used qualitative data collection methods, such as interviewing, for item generation. Limited data on the process of psychometric evaluation usually prevents researchers from choosing the most appropriate tool. Most studies had reported construct validity assessment.

5. Which concept is measured by the tool?

The concept of hospital preparedness can encompass three sub-concepts including structural, non-structural, and functional preparedness 20. Our findings revealed that most of the reviewed tools mainly dealt with structural and non-structural preparedness and despite its paramount importance, most of the tools did not measure the functional aspect of HDP. The reason for this shortcoming is the fact that the concept of HDP has not been systematically analyzed and explored yet. In other words, the definition of the concept and its attributes remain unclear 34.

6. Has the tool been developed by using a theoretical framework?

A key component of instrument development guidelines is using a theoretical framework for developing the intended tool 35. The American Psychological Association clearly stated that the construct which is going to be measured needs to be explained in a theoretical framework. As numerous concepts are related to the construct of HDP, the tools which are going to measure this construct need to be developed by using an appropriate theoretical framework. However, none of the reviewed studies had reported using a sound theoretical framework for developing their tools. We found that most of the tools simply measured the vulnerability of hospitals and hence, the concept of HDP has been taken for granted. Future studies primarily need to develop a sound theoretical framework and then use it for developing tools that could measure the concept.

7. How is it completed?

Is it a self-report tool or should it be completed by conducting interview? When a tool is completed by using the interview method, ambiguities, if any, are clarifiedand none of the items are to be left unanswered 35. We found that the application of most HDP tools is time-consuming and costly. For instance, the best sources for acquiring information on the risk assessment domain of the HIS are agricultural and meteorological organizations. Moreover, this domain should be completed by qualified technical experts. Accordingly, this tool should be completed through team work. However, some tools such as ACEP were completed on a self-report basis.

8. Is it completed easily and conveniently?

Study findings revealed that the completion of tools which assess structural HDP—such as HSI, Mulyasari, CMS, APIC, AHRO, TDH, and VHA—is difficult, needs team work, and takes considerable amount of time.

9. Are complex statistical analyses needed for calculating the score of the tool?

None of the reviewed tools necessitate complex statistical analyses for calculating the final score. However, the results of most tools are presented on a categorical scale (for example, ‘Excellent, Good, Moderate, Poor, unacceptable’, ‘Yes, No’, or ‘Low, Moderate, High’) and hence, it is difficult to identify the exact line between preparedness and unpreparedness when using these tools. Quantification of these tools can improve their clarity and applicability.

10. Is it a context-bound tool or an international one?

The results of our review showed that despite the great impacts of cultural issues on HDP, particularly on the functional preparedness domain, none of the current HDP tools have been developed contextually.

## Conclusion

Study findings indicate that because the HDP tools have not been developed through the process of psychometric evaluation, some indicators of HDP might have remained neglected. HDP can be evaluated by valid and reliable tools which are developed in careful methodological studies through psychometric evaluation process 12. Study findings also showed that currently, there is no comprehensive tool for evaluating HDP. One of the weaknesses of the current tools is their inability to evaluate all aspects of HDP including; structural, non-structural and functional. These tools mainly focus on the structural and non-structural HDP and scarcely deal with the functional HDP 36. The other weakness is related to pitfalls in their psychometric evaluation and/or theoretical frameworks. Moreover, most of the reviewed tools had not been developed based on empirical data. Healthcare systems need evaluation tools which are developed by using experts’ knowledge and experience and are validated through the process of psychometric evaluation.

This systematic review study evaluated all general and specific HDP tools. The aim of systematic reviews is not to exactly determine the accuracy and the precision of the available tools. Consequently, we did not evaluate the accuracy and the precision of the reviewed tools.

**Recommendations and** **Implications**

HDP is a long chain that consists of multiple loops. Therefore, it is important to ensure the appropriate tool is used to assess HDP enabling an acceptable response when encounteringto disasters.

Scrutiny of HDP tools, can help researchers in identification of strengths and weaknesses of existent tools, and to take steps to develop the proper tools to survey the level of HDP.

Given the weaknesses of the current HDP tools, further mixed-methods and qualitative studies are needed for exploring and clarifying the concept of HDP and developing comprehensive tools which assess the functional aspects of HDP.

## Limitation

The main limitation of this review was that only English language papers included in the study as a systematic Review. Therefore, we lost some of the relevant studies which were not in English language.

## Appendix: PRISMA 2009 Checklist for "Hospital Disaster Preparedness tool: A Systematic Review"

**Table:** 

Section/topic	#	Checklist item	Reported on page #
TITLE			
Title	1	Identify the report as a systematic review, meta-analysis, or both.	1
ABSTRACT			
Structured summary	2	Provide a structured summary including, as applicable: background; objectives; data sources; study eligibility criteria, participants, and interventions; study appraisal and synthesis methods; results; limitations; conclusions and implications of key findings; systematic review registration number.	1
INTRODUCTION			
Rationale	3	Describe the rationale for the review in the context of what is already known.	1
Objectives	4	Provide an explicit statement of questions being addressed with reference to participants, interventions, comparisons, outcomes, and study design (PICOS).	2
METHODS			
Protocol and registration	5	Indicate if a review protocol exists, if and where it can be accessed (e.g., Web address), and, if available, provide registration information including registration number.	2
Eligibility criteria	6	Specify study characteristics (e.g., PICOS, length of follow-up) and report characteristics (e.g., years considered, language, publication status) used as criteria for eligibility, giving rationale.	2-3
Information sources	7	Describe all information sources (e.g., databases with dates of coverage, contact with study authors to identify additional studies) in the search and date last searched.	3
Search	8	Present full electronic search strategy for at least one database, including any limits used, such that it could be repeated.	2-3
Study selection	9	State the process for selecting studies (i.e., screening, eligibility, included in systematic review, and, if applicable, included in the meta-analysis).	3
Data collection process	10	Describe method of data extraction from reports (e.g., piloted forms, independently, in duplicate) and any processes for obtaining and confirming data from investigators.	3
Data items	11	List and define all variables for which data were sought (e.g., PICOS, funding sources) and any assumptions and simplifications made.	4
Risk of bias in individual studies	12	Describe methods used for assessing risk of bias of individual studies (including specification of whether this was done at the study or outcome level), and how this information is to be used in any data synthesis.	2
Summary measures	13	State the principal summary measures (e.g., risk ratio, difference in means).	N/A
Synthesis of results	14	Describe the methods of handling data and combining results of studies, if done, including measures of consistency (e.g., I^2^) for each meta-analysis.	4

**Table:** 

Section/topic	#	Checklist item	Reported on page #
Risk of bias across studies	15	Specify any assessment of risk of bias that may affect the cumulative evidence (e.g., publication bias, selective reporting within studies).	3
Additional analyses	16	Describe methods of additional analyses (e.g., sensitivity or subgroup analyses, meta-regression), if done, indicating which were pre-specified.	N/A
RESULTS			
Study selection	17	Give numbers of studies screened, assessed for eligibility, and included in the review, with reasons for exclusions at each stage, ideally with a flow diagram.	3
Study characteristics	18	For each study, present characteristics for which data were extracted (e.g., study size, PICOS, follow-up period) and provide the citations.	3-7-8-9-10-11
Risk of bias within studies	19	Present data on risk of bias of each study and, if available, any outcome level assessment (see item 12).	N/A
Results of individual studies	20	For all outcomes considered (benefits or harms), present, for each study: (a) simple summary data for each intervention group (b) effect estimates and confidence intervals, ideally with a forest plot.	3
Synthesis of results	21	Present results of each meta-analysis done, including confidence intervals and measures of consistency.	N/A
Risk of bias across studies	22	Present results of any assessment of risk of bias across studies (see Item 15).	3
Additional analysis	23	Give results of additional analyses, if done (e.g., sensitivity or subgroup analyses, meta-regression [see Item 16]).	N/A
DISCUSSION			
Summary of evidence	24	Summarize the main findings including the strength of evidence for each main outcome; consider their relevance to key groups (e.g., healthcare providers, users, and policy makers).	3-4-5
Limitations	25	Discuss limitations at study and outcome level (e.g., risk of bias), and at review-level (e.g., incomplete retrieval of identified research, reporting bias).	6
Conclusions	26	Provide a general interpretation of the results in the context of other evidence, and implications for future research.	5
FUNDING			
Funding	27	Describe sources of funding for the systematic review and other support (e.g., supply of data); role of funders for the systematic review.	1

*From: * Moher D, Liberati A, Tetzlaff J, Altman DG, The PRISMA Group (2009). Preferred Reporting Items for Systematic Reviews and Meta-Analyses: The PRISMA Statement. PLoS Med 6(6): e1000097. doi:10.1371/journal.pmed1000097

For more information, visit: **www.prisma** **-** **statement.org**.

## Correspondence 

 Corresponding author: Dr Abbas Ebadi. Email: ebadi1347@yahoo.com
